# Phosphorus/Phosphide‐Based Materials for Alkali Metal‐Ion Batteries

**DOI:** 10.1002/advs.202200740

**Published:** 2022-04-09

**Authors:** Fangzheng Chen, Jie Xu, Shanying Wang, Yaohui Lv, Yang Li, Xiang Chen, Ailin Xia, Yongtao Li, Junxiong Wu, Lianbo Ma

**Affiliations:** ^1^ Low‐Carbon New Materials Research Center Low‐Carbon Research Institute, School of Materials Science and Engineering Anhui University of Technology Maanshan 243002 China; ^2^ Key Laboratory of Green Fabrication and Surface Technology of Advanced Metal Materials Ministry of Education Maanshan 243002 China; ^3^ Department of Mechanical and Aerospace Engineering The Hong Kong University of Science and Technology (HKUST) Clear Water Bay Hong Kong 999077 China; ^4^ College of Environmental Science and Engineering Fujian Normal University Fuzhou Fujian 350000 China

**Keywords:** alkali metal‐ion batteries, anodes, electrochemical energy storage, phosphide, phosphorus

## Abstract

Phosphorus‐ and phosphide‐based materials with remarkable physicochemical properties and low costs have attracted significant attention as the anodes of alkali metal (e.g., Li, Na, K, Mg, Ca)‐ion batteries (AIBs). However, the low electrical conductivity and large volume expansion of these materials during electrochemical reactions inhibit their practical applications. To solve these problems, various promising solutions have been explored and utilized. In this review, the recent progress in AIBs using phosphorus‐ and phosphide‐based materials is summarized. Thereafter, the in‐depth working principles of diverse AIBs are discussed and predicted. Representative works with design concepts, construction approaches, engineering strategies, special functions, and electrochemical results are listed and discussed in detail. Finally, the existing challenges and issues are concluded and analyzed, and future perspectives and research directions are given. This review can provide new guidance for the future design and practical applications of phosphorus‐ and phosphide‐based materials used in AIBs.

## Graphical abstract

Alkali metal‐ion batteries (AIBs) using phosphorus‐ and phosphide‐based materials have been considered as the mainstream energy storage and conversion devices. This review summarizes the recent progress on AIBs using phosphorus‐ and phosphide‐based materials, analyzes their design concepts, construction strategies, special functions, and electrochemical results, and it is believed to supply important guidance for their further developments and practical applications.

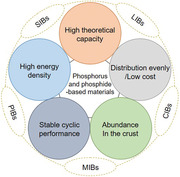

## 1 Introduction

With the depletion of fossil resources and the intensification of greenhouse effect, energy crisis has become a crucial worldwide problem.^[^ ^1^, ^2^, ^3^, ^4^, ^5^, ^6^, ^7^ ^]^ As alternatives to conventional fossil fuels, sustainable and renewable energy sources, including solar, geothermal, tidal, wind, and water energies have attracted significant attention;^[^ ^8^, ^9^, ^10^ ^]^ however, disadvantages such as uneven distribution, weather impact, and unstable energy power, severely restrict their extensive applications.^[^ ^11^, ^12^ ^]^ In the past few decades, rechargeable batteries with sustainable energy supply, high specific capacity, stable cyclic performance, and low cost were widely applied to store the above energy resources.^[^ ^13^, ^14^ ^]^ Particularly, lithium‐ion batteries (LIBs) have been widely used in hybrid electric vehicles and portable electronic products owing to their ultra‐long lifetime.^[^ ^15^, ^16^, ^17^, ^18^, ^19^ ^]^ Recently, with the rapid development of high‐end electronic markets,^[^ ^20^, ^21^ ^]^ other alkali metal‐ion batteries (AIBs) including sodium‐ion batteries (SIBs), potassium‐ion batteries (PIBs), and multivalent‐ion batteries have been considered as the next‐generation energy power.^[^ ^22^ ^]^ However, similar to that of LIBs, the anodes of these AIBs encounter various crucial issues, such as poor electrical conductivity, low redox potential, severe volume expansion, and limited capacity/energy density, hampering their actual utilizations.^[^ ^23^ ^]^

To solve the above issues of AIBs, many efforts have been devoted into the exploration of new anodes with novel architectures. To date, the emerged anode materials include carbonaceous materials,^[^ ^24^, ^25^ ^]^ elementary substance (e.g., Sn, Sb, Bi, and P),^[^ ^26^, ^27^, ^28^, ^29^, ^30^ ^]^ and metal compounds (e.g., metal oxides, sulfides, selenides, phosphides, and nitrides).^[^ ^31^, ^32^, ^33^, ^34^, ^35^, ^36^, ^37^, ^38^ ^]^ Carbonaceous materials have excellent electrical conductivity, but their theoretical capacity is relatively low. Conversely, elementary substance and metal compounds have high theoretical capacity, but they suffer from slow electron transfer and low reaction kinetics. By comparing the advantages and disadvantages of anode materials, elementary substance and metal compounds should be the suitable candidates to be used in AIBs, and great electrochemical results have been achieved. Particularly, phosphorus‐ and phosphide‐based materials have attracted significant attention because of the high theoretical capacity (phosphorus: 2596 mAh g^−1^, based on the formation of M_3_P), low redox potential, and high reserve abundance in the earth.^[^ ^39^ ^]^ However, these materials experience severe volume expansion and low reaction kinetics during cycles, which degrades their electrochemical performance.

Phosphorus mainly has three allotropes: white phosphorus (WP), red phosphorus (RP), and black phosphorus (BP).^[^ ^39^ ^]^ Of them, BP is the most stable form and possesses a graphite‐like layered structure, indicating a good electrical conductivity. Initially, it was revealed that BP exhibited a capacity of 1300 mAh g^−1^ in LIBs,^[^ ^40^ ^]^ which is significantly higher than that of graphite (372 mAh g^−1^). Thus, more attention has been focused on phosphorus‐based architectures. For instance, Zhou et al. fabricated hollow RP nanospheres (HPNs) with porous shells as the anode materials of LIBs.^[^ ^41^ ^]^ This structure possesses enough space for accommodating the large volume expansion; moreover, the porous channels are beneficial to the ion diffusion and electrolyte penetration, enhancing the electrochemical results. This study demonstrated that reducing the particle size of phosphorus or constructing hollow/porous structures were efficient strategies. Incorporating pure phosphorus‐based materials with other materials including carbonaceous materials, polymers, and metal compounds is another useful strategy for overcoming the issues of AIBs. For instance, Wang et al. prepared RP nanoparticles wrapped in reduced graphene oxide (rGO) matrix as the anode material of PIBs.^[^ ^42^ ^]^ rGO containing residual oxygen atoms and multiple active sites increases the structural disorder, reduces the energy barrier of chemical reactions, and generates P—C bonds, contributing to a good electrochemical result.

In addition to pure phosphorus‐based materials, phosphide‐based materials can be employed as the anodes of AIBs. To date, the emerged metal phosphides include FeP*_x_*,^[^ ^43^ ^]^ CoP*_x_*,^[^ ^44^ ^]^ NiP*_x_*,^[^ ^45^ ^]^ and SnP*_x_*,^[^ ^46^, ^47^ ^]^ and these anode materials demonstrated excellent electrochemical results. Of them, SnP*_x_* has recently attracted significant interests, owing to the ultra‐high capacity according to the alloy reactions between alkali metal ions and Sn/P elements.^[^ ^46^, ^47^ ^]^ For instance, Zhao et al. constructed hollow yolk‐shell Sn_4_P_3_ nanospheres as anode material of SIBs.^[^ ^48^ ^]^ Benefiting from the above special structures, Sn_4_P_3_ nanospheres delivered a highly reversible discharge capacity of 297.6 mAh g^−1^. However, similar to pure phosphorus, phosphides suffered from low electrical conductivity and significant volume expansion. Hybridizing phosphides with carbonaceous materials can solve the above issues, and the adopted carbonaceous materials include graphene,^[^ ^49^ ^]^ carbon nanotubes (CNTs),^[^ ^50^ ^]^ and porous carbon matrices.^[^ ^51^ ^]^ For instance, Zhao et al. immobilized Ni_2_P into carbon nanosheets (CNSs) and combined FeP quantum dots into CNT‐P‐carbon octahedra, respectively, and employed them as anode materials of SIBs.^[^ ^51^, ^52^ ^]^ It was revealed that these carbon matrices effectively inhibited the aggregation of phosphide nanoparticles, enhanced the electronic conductivity, and maintained a high structural integrity.

To enhance the real‐world applications of phosphorus‐ and phosphide‐based materials from lab stage to commercialization, a systematic and comprehensive review is urgently needed to provide useful guidance for their further exploration and utilization in AIBs. In this review, the recent progress on AIBs using phosphorus‐ and phosphide‐based materials are summarized. Subsequently, the working principles of diverse AIBs and challenges facing phosphorus‐ and phosphide‐based materials are discussed. Thereafter, representative works for different AIBs are listed and analyzed, based on design concepts, preparation methods, engineering strategies, structural tuning, and electrochemical results. Finally, the remaining challenges/issues are concluded and discussed, and future perspectives as well as research directions are given.

## 2 Working Principles and Challenges of Phosphorus‐ and Phosphide‐Based Materials in AIBs

**Figure 1:**
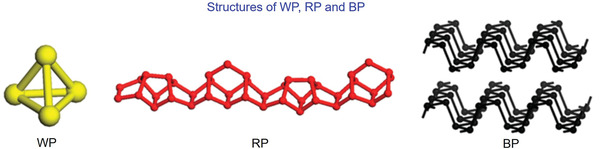
Schematic illustrations of the typical structures of WP, RP, and BP. Reproduced with permission.^[^ ^53^ ^]^ Copyright 2014, American Chemical Society.

Phosphorus can be mainly divided into WP, RP, and BP, and their typical structures are shown in **Figure** **1**.^[^ ^53^ ^]^ Of them, RP is the most extensively investigated material in AIBs, although it has a moderate electrical conductivity. The main working principle of phosphorus‐based materials in LIBs, SIBs, and PIBs is the alloying/dealloying mechanism,^[^ ^22^ ^]^ and it is responsible for most of the capacity values of AIBs. Regarding LIBs and SIBs, the alloying products were Li_3_P and Na_3_P,^[^ ^54^, ^55^ ^]^ whereas for PIBs, the final alloying products included KP, K_3_P, and K_4_P_3_,^[^ ^56^, ^57^, ^58^ ^]^ based on different working pathways. Notably, BP possesses a 2D layer structure, thus the intercalation process also contributed to partial capacity value of AIBs.^[^ ^55^ ^]^

Phosphide‐based materials usually have numerous compositions, morphologies, and structures, and thus the working principles varied significantly. Normally, the working principles included intercalation/deintercalation, chemical conversion, and alloying/dealloying mechanisms. Intercalation/deintercalation mechanism is the simplest strategy for generating capacity, and the typical characteristic is the intercalation of alkali metal ions into/out the main structure of phosphide‐based materials.^[^ ^59^ ^]^ Typically, Nazar et al. reported the low‐potential intercalation/deintercalation of Li^+^ into MnP_4_ structure, generating an electrochemical reaction of MnP_4_ to Li_7_MnP_4_.^[^ ^60^ ^]^ By far, other emerged phosphides that followed intercalation/deintercalation mechanism included manganese phosphide (MnP)^[^ ^61^ ^]^ and vanadium phosphide (VP).^[^ ^62^ ^]^ For chemical conversion mechanism, it normally induces a better alkali metal ion storage performance. For metal phosphides (M*_x_* P), phosphorus element reacts with alkali metal ions, accompanying the generation and uniform distribution of pure M species.^[^ ^59^ ^]^ Notably, M species are beneficial to the electrochemical performance of AIBs, preventing structural pulverizations and promoting fast electron transfer. Copper phosphide (Cu_3_P),^[^ ^63^ ^]^ nickel phosphide (Ni_2_P),^[^ ^64^, ^65^ ^]^ iron phosphide (FeP),^[^ ^66^, ^67^, ^68^ ^]^ molybdenum phosphide (MoP),^[^ ^69^, ^70^ ^]^ and vanadium phosphide (V_4_P_7_)^[^ ^71^ ^]^ were verified to follow the chemical conversion mechanism, and of them, FeP showed the great potential in practical applications, because of the achieved high capacity and long‐term cyclic stability in SIBs.^[^ ^72^ ^]^ Lastly, alloying/dealloying mechanism is similar to that in phosphorus‐based materials, and this mechanism usually contributes the highest capacity values for AIBs. Different from that in chemical conversion mechanism, the typical feature of alloying/dealloying mechanism is the alloying of alkali metal ions (A*^y^* ^+^) with both M and phosphorus, thus forming the final products of A*_m_* M and A*_n_* P. The emerged phosphide‐based materials followed this mechanism were tin phosphide (Sn_4_P_3_),^[^ ^73^, ^74^, ^75^ ^]^ germanium phosphide (GeP_5_),^[^ ^76^, ^77^ ^]^ silicon phosphide (SiP_2_),^[^ ^78^ ^]^ and zinc phosphide (Zn_3_P_2_),^[^ ^79^ ^]^ and specifically, Sn_4_P_3_ usually exhibited the highest electrochemical performance in AIBs.^[^ ^80^ ^]^

Since phosphorus‐ and phosphide‐based materials exhibited diverse working mechanisms in AIBs, issues, and challenges were usually produced, mainly including slow reaction kinetics and serious volume changes. Phosphorus and phosphides intrinsically showed low electronic conductivity,^[^ ^81^ ^]^ and this restricted the fast electron transfer during electrochemical reactions, thereby generating slow reaction kinetics. Moreover, the low electronic conductivity may decrease the utilization ratio of active materials, leading to rapid capacity decay.^[^ ^39^ ^]^ The other main challenge facing phosphorus‐ and phosphide‐based materials is their serious volume changes during cycles. Upon discharging, phosphorus‐ and phosphide‐based materials suffered from an obvious volume change when reacted with alkali metal ions,^[^ ^39^, ^82^ ^]^ this would cause the severe structural damage/collapse, and even pulverization during repeated cycles. Impressively, the serious volume changes would induce the continuous generation and broken of solid electrolyte interface (SEI) layers,^[^ ^39^, ^40^ ^]^ thus consuming the electrolyte and degrading the cyclic stability and Coulumbic efficiency (CE).^[^ ^41^, ^46^ ^]^ To resolve these pressing issues, the promising strategies included the hybridization of phosphorus‐ and phosphide‐based materials with conductive materials (e.g., carbonaceous materials and conductive polymers) and constructing unique micro‐ and nanostructures,^[^ ^42^, ^49^ ^]^ which can boost the electron transfer, provide enough space for volume changes and enhance the entire structural integrity of electrode.

Therefore, compared with those AIBs using phosphorus‐ and phosphide‐based materials, there are some similarities and differences in working mechanisms, electrochemical performance, and challenges: 1) LIBs, SIBs, and PIBs show almost the similar working mechanisms when using phosphorus‐ and phosphide‐based electrodes, but the reaction kinetics and ion diffusion speeds, may be different because of the different ion radius of Li^+^, Na^+^, and K^+^. Moreover, the reaction pathways and generated intermediates may be different, thus contributing to different theoretical capacities for different battery types. 2) LIBs, SIBs, and PIBs display remarkable electrochemical performance when adopted phosphorus‐ and phosphide‐based electrodes, however, the electrochemical performance varied significantly owing to the different working mechanisms and ion radius. 3) LIBs, SIBs, and PIBs face many future challenges when using phosphorus‐ and phosphide‐based electrodes, such as limited long‐term cyclic stability, side reactions, metal dendrite growth, and safety concerns. For LIBs, the main obstacles should be metal dendrite growth and relatively low discharge capacity, while for SIBs and PIBs, the main issues include metal dendrite growth, side reactions, limited cyclic stability, and safety problem, which makes SIBs and PIBs more difficult for manipulation and further commercialization.

## 3 Phosphorus‐ and Phosphide‐Based Materials for LIBs

Although phosphorus‐ and phosphide‐based materials possess diverse advantages in LIBs,^[^ ^83^, ^84^ ^]^ they are normally restricted by several pressing issues, as discussed in Section 2. In the following sections, these discussions will be divided into phosphorus‐based materials and phosphide‐based materials (**Table** **1**), from the viewpoints of elaborately constructing micro‐and nanostructures (e.g., hollow, porous, yolk‐shell) and introducing conductive additives.

**Table 1:** Phosphorus‐ and phosphide‐based materials for LIBs

Materials	Morphology	Synthesis method	Rate performance	Cyclic performance	Ref.
P‐SCNT	Porous structure	Hydrothermal method	800 mAh g^−1^ at 2.0 A g^−1^	1400 mAh g^−1^ after 200 cycles at 0.1 A g^−1^	[91]
P/CNG	3D skeleton structure	Ball‐milling	1340 mAh g^−1^ at 3.9 A g^−1^	1470 mAh g^−1^ after 300 cycles at 1.3 A g^−1^	[99]
P‐PCNFs	Porous carbon nanofiber structure	Vaporization–adsorption–transformation	1673 mAh g^−1^ at 2.6 A g^−1^	850 mAh g^−1^ after 100 cycles at 0.26 A g^−1^	[104]
Ni_2_P@C‐CNTs	Cubic nanoparticle structure	Vaporization–condensation	755 mAh g^−1^ at 5.0 A g^−1^	654 mAh g^−1^ after 1500 cycles at 5.0 A g^−1^	[109]
Ni_12_P_5_@carbon fiber	Peapod‐like structure	Hydrothermal method	380 mAh g^−1^ at 3.0 A g^−1^	660 mAh g^−1^ after 100 cycles at 0.1 A g^−1^	[112]
Fe_2_P/GC	Yolk/shell octahedra structure	Hydrothermal method and calcination	392 mAh g^−1^ at 10.0 A g^−1^	612 mAh g^−1^ after 200 cycles at 0.1 A g^−1^	[114]
FeP@carbon	Porous nanoplate structure	Hydrothermal reaction and phosphidation	347 mAh g^−1^ at 5.0 A g^−1^	610 mAh g^−1^ after 400 cycles at 0.5 A g^−1^	[116]
Co_2_P/graphene	Porous structure	One‐pot solution method	312 mAh g^−1^ at 1.6 A g^−1^	901 mAh g^−1^ after 250 cycles at 0.1A g^−1^	[118]
Co*_x_* P@NC	Porous nanowire structure	Carbonization and phosphidation	256 mAh g^−1^ at 2.0 A g^−1^	526 mAh g^−1^ after 700 cycles at 1.0 A g^−1^	[119]
GaP@TiO_2_‐carbon black	Porous structure	High‐energy mechanical milling	695 mAh g^−1^ at 10 A g^−1^	1012 mAh g^−1^ after 500 cycles at 0.5 A g^−1^	[124]

### 3.1 Phosphorus‐Based Materials for LIBs

#### 3.1.1 Pure Phosphorus

Phosphorus can be employed as anode materials for LIBs. Using RP and WP as raw materials, Sun et al. synthesized WBP (BP obtained from WP) and RBP (BP obtained from RP) as the anodes of LIBs, through a high‐temperature and high‐pressure method.^[^ ^85^ ^]^ They revealed that the electrochemical activity of RBP is better than that of WBP, and the maximum discharge/charge capacities were 2649 and 1425 mAh g^−1^, respectively. However, after merely 60 cycles, the capacity of RBP anode decreased rapidly to 703 mAh g^−1^, owing to the crushing and rupture of active materials induced by large volume expansion during cycles. To address these issues, controllably adjusting the morphology and detailed structure of phosphorus is a facile and effective strategy. Notably, phosphorus‐based materials prepared using physical methods were verified to hardly meet the requirements, because of the simple structures and large particle sizes. In 2017, Zhou et al. firstly employed a solvothermal approach to fabricate hollow RP nanospheres (HPNs) with porous shells.^[^ ^41^ ^]^ The structure has enough space for accommodating the large volume expansion, and porous channels for facilitating ion diffusion; thereby, HPNs electrode with a high RP percentage (60 wt.%) showed excellent long‐life cyclic stability, with a capacity of 1048 mAh g^−1^ after 600 cycles at 2.6 A g^−1^.

#### 3.1.2 Phosphorus/Carbon Hybrids

##### Phosphorus/Carbon Black and Phosphorus/Graphite

The hybridization of conductive carbonaceous materials with phosphorus can take full advantage of their intrinsic merits including high electronic conductivity, large surface area, good mechanic properties (originated from carbonaceous materials), and high theoretical capacity (mainly derived from phosphorus). Toward this goal, carbon black was utilized as the carbon additive.^[^ ^82^ ^]^ Typically, Park et al. reported the combination of BP and super P (carbon black) using high‐energy mechanical milling, and they employed it as the anode for LIBs.^[^ ^86^ ^]^ An excellent electrochemical result including high initial CE (90%) and good cyclic performance (600 mAh g^−1^ after 100 cycles at 100 mA g^−1^) was achieved. By adopting graphite as carbon matrix, carbon atoms connected well with phosphorus atoms, forming stable C—P bonds. Sun et al. investigated the effects of BP‐graphite (composite) and BP/graphite (hybrid) on battery performance.^[^ ^53^ ^]^ It was disclosed that P—C bond was generated when using BP and graphite as starting materials, ensuring the effective contact between phosphorus and carbon material. As a result, the discharge capacity of the as‐prepared anodes is 2786 mAh g^−1^ at 520 mA g^−1^, and the capacity retention is as high as 80% after 100 cycles. Moreover, Ramireddy et al. used ball milling method to hybridize BP with graphite,^[^ ^87^ ^]^ and the as‐achieved anodes exhibited a high initial capacity of 1700 mAh g^−1^, and then it decreased to 349 mAh g^−1^ after merely 50 cycles. The difference in electrochemical results may be attributed to the difficulties in controlling the particle size and morphology of phosphorus/carbon hybrids. However, these achieved electrochemical performance is still low, and can be further improved. Hybridizing phosphorus/graphite further with active carbon material is a promising solution, the generated graphite/P@active carbon anode exhibited an enhanced electrochemical performance (500 mAh g^−1^ at 260 mA g^−1^, 485 mAh g^−1^ after 50 cycles at 260 mA g^−1^).^[^ ^88^ ^]^

##### Phosphorus/CNTs

**Figure 2:**
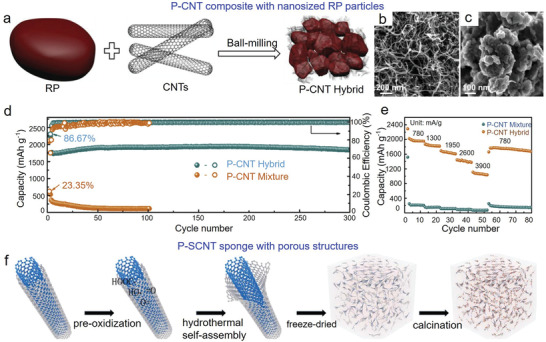
Phosphorus/CNT hybrids used in LIBs. a) Schematic illustration of the synthesis of P‐CNT hybrid. SEM images of b) CNT and c) P‐CNT hybrid. d,e) Cyclic performance and rate capability of P‐CNT hybrid and P‐CNT mixture. Reproduced with permission.^[^ ^89^ ^]^ Copyright 2019, Elsevier. f) Schematic illustration of the synthesis of P‐SCNT composite. Reproduced with permission.^[^ ^91^ ^]^ Copyright 2018, Elsevier.

CNTs are seamless hollow tubes made of single‐ or multi‐layered graphite sheets. They normally have excellent electronic conductivity and good mechanical property; and can combine with phosphorus to form hybrid architectures. The simplest method for preparing P/CNT composite is high‐energy ball milling.^[^ ^89^, ^90^ ^]^ For instance, Jiao et al. prepared a novel type of P‐CNT hybrid via this approach, bulk phosphorus and CNTs converted into nano‐sized particles, and they were evenly distributed within the hybrid (**Figure** **2**a–c).^[^ ^89^ ^]^ During ball milling process, P—O—C chemical bond was formed between RP and CNTs; thus a close contact was generated, improving the electronic conductivity and cyclic performance. The as‐prepared P‐CNT hybrid exhibited a capacity of 2252 mAh g^−1^, and maintained at 1844 mAh g^−1^ even after 300 cycles (Figure 2d,e). However, the ball milling method usually produced phosphorus/CNTs with large particle size and uncontrollable structures. To date, other promising strategies have been emerged. For instance, Yuan et al. restricted RP within 3D sheared CNT (SCNT) conductive sponge (P‐SCNT) (Figure 2f).^[^ ^91^ ^]^ The 3D SCNT aerogel sponge has intrinsically coupled units, the inside comprises of 1D single‐ or few‐walled CNTs, and the outside is composed of 2D sheared single carbon layer, endowing it with large surface area and high porosity. This not only improves the electronic conductivity of phosphorus, but also buffers the structural strain/stress‐induced by volume changes. Therefore, in comparison with P/SCNT mixture, the as‐prepared P‐SCNT anode exhibited significantly improved electrochemical results, including a reversible capacity of 1600 mAh g^−1^ at 100 mA g^−1^, and good cyclic stability with a capacity of 1400 mAh g^−1^ after 200 cycles at 100 mA g^−1^.

To enhance the electrochemical results of RP/CNT composite further, Sun et al. loaded TiO_2_ onto the surface of P/CNT (P‐TiO_2_@CNT) using a simple solution‐based method.^[^ ^92^ ^]^ As buffer materials, TiO_2_ bonded with RP and accommodated the generated stress upon lithiation, while CNTs served as conductive channels and alleviated volume changes. As a result, the as‐prepared P‐TiO_2_@CNT anode exhibited a high reversible capacity of 1530 mAh g^−1^ and high‐rate capability (750 mAh g^−1^ at 10 A g^−1^).

##### Phosphorus/Graphene

**Figure 3:**
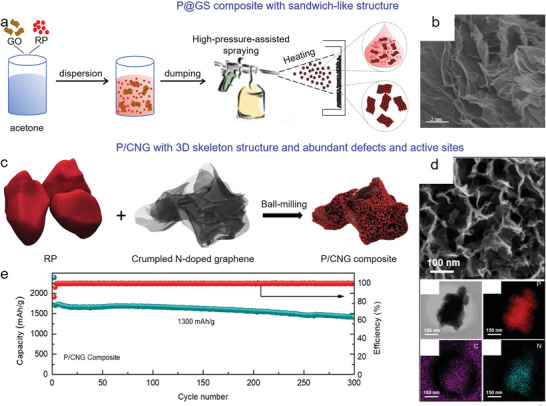
Phosphorus/graphene composites used in LIBs. a) Schematic illustration of the preparation of sandwich‐like P@GS hybrid, and b) the typical morphology. Reproduced with permission.^[^ ^94^ ^]^ Copyright 2016, Elsevier. c) Schematic illustration of the preparation of P/CNG. d) Typical morphology and element mapping of P/CNG composite. e) Long‐term cyclic performance of P/CNG composite. Reproduced with permission.^[^ ^99^ ^]^ Copyright 2019, American Chemical Society.

Graphene, a 2D carbon CNS with high electronic conductivity, large surface area (theoretically 2630 m^2^ g^−1^), and good mechanical flexibility, has attracted significant attention in energy‐related fields.^[^ ^22^ ^]^ Combining phosphorus with graphene‐enhanced the utilization ratio and electrochemical results of phosphorus‐based anodes in LIBs. For instance, Yu et al. prepared RP‐graphene nanosheet (P—G) hybrid as the anode of LIBs.^[^ ^93^ ^]^ It was revealed that graphene layer hybridized with phosphorus particles formed a 3D conductive integrated network, and the presence of P—O—C bonds in hybrid maintained the contact between RP and graphene network. Consequently, the as‐prepared anodes delivered a high initial capacity of 2517 mAh g^−1^, and the capacity retention was 60% even after 300 cycles at 260 mA g^−1^. Moreover, Wang et al. employed the high‐pressure assisted spraying method to synthesize a unique sandwich‐like P@GS hybrid, of which nanosized phosphorus particles were stacked onto rGO nanosheets (GS).^[^ ^94^ ^]^ Electrochemical results revealed a high initial discharge capacity of 1876 mAh g^−1^, high capacity retention of 990 mAh g^−1^ after 50 cycles, and great rate capability with a capacity of 700 mAh g^−1^ at 1.0 A g^−1^ (**Figure** **3**a,b). In addition, other BP/rGO and RP/rGO hybrids were fabricated through various strategies,^[^ ^95^, ^96^, ^97^ ^]^ and they all exhibited good electrochemical results. However, these investigations also suffered from the low utilization ratio and volume expansion of phosphorus, degrading the achieved capacity values.

To address the abovementioned issues, Yan et al. prepared a 3D conductive carbon scaffold (ACW) using natural wood as precursor, and then loaded P@rGO on ACW surface through a vaporization‐condensation method.^[^ ^98^ ^]^ This structure suppressed the volume expansion of phosphorus, enabled a high phosphorus content, and promoted electron/ion transfer. Moreover, Jiao et al. designed a P/crumpled N‐doped graphene (P/CNG) composite (Figure 3c,d).^[^ ^99^ ^]^ Owing to the 3D skeleton structure and abundant defects as well as active sites generated in CNG sheet, the serious volume change of RP was well relieved. As a result, P/CNG anode exhibited a high‐rate capability (1340 mAh g^−1^ at 3.9 A g^−1^) and good cyclic performance with a capacity of 1470 mAh g^−1^ after 300 cycles at 1.3 A g^−1^ (Figure 3e). Similarly, Shi et al. fabricated a BP/G composite for LIBs,^[^ ^100^ ^]^ and a high electrochemical result (initial capacity of 1962 mAh g^−1^ at 260 mA g^−1^, and then maintained at 1514 mAh g^−1^ after 100 cycles) was realized.

##### Phosphorus/Porous Carbon Matrices

Porous carbon architectures possess the apparent structural merits including large surface area, accessible pores, voids, and channels, as well as high structural integrity, facilitating the efficient contact with the electrolyte and further shortening ion diffusion distance.^[^ ^101^ ^]^ Moreover, porous carbon architectures with enough voids and space accommodated volume expansion and enabled a high content of phosphorus. For instance, Wang et al. encapsulated RP into porous carbon using a simple vaporization/adsorption strategy,^[^ ^102^ ^]^ and electrochemical testing revealed a high capacity of 800 mAh g^−1^ at 100 mA g^−1^. This work confirmed that porous carbon improved the electrochemical activity of RP in LIBs. In addition, Wang et al. employed a similar method to construct the hybrid comprising of amorphous RP and porous carbon,^[^ ^103^ ^]^ and a high reversible capacity of 1550 mAh g^−1^ was achieved. The high electrochemical results can be attributed to the uniform distribution of amorphous RP within the micropores of carbon frameworks and high RP content (60.0 wt.%).

To promote the utilization of phosphorus‐based materials in LIBs further, intriguing micro‐and nanostructures were desired, because they can satisfy almost all requirements. For instance, Li et al. embedded RP into porous carbon nanofibers (PCNFs) to form a 3D flexible composite for LIBs.^[^ ^104^ ^]^ PCNFs effectively accommodated the volume expansion of RP during cycles, and accelerated ion diffusion. Recently, Yan et al. prepared a porous carbon skeleton by pyrolyzing ZIF‐8, and then embedded the blocky RP into the pores/voids of carbon skeletons.^[^ ^105^ ^]^ This method avoided the surface phosphorus crystallization and generation of WP; thus the volume expansion of phosphorus was well alleviated. Eventually, a capacity of 400 mAh g^−1^ after 700 cycles at 1.0 A g^−1^ was obtained, indicating an excellent cyclic stability.

#### 3.1.3 Phosphorus/Conductive Polymers

Similar to carbonaceous materials, conductive polymers have also been adopted to enhance the electrical conductivity of phosphorus and further alleviate its volume expansion. For instance, Baboukani et al. developed a binder‐free RP/sulfurized polyacrylonitrile (RP/SPAN) hybrid using the ball milling method combined with an electrostatic spray deposition process.^[^ ^106^ ^]^ SPAN connected well with RP by forming efficient chemical bonds, and it served as the conductive agent for improving the reaction kinetics. When used as the anodes of LIBs, a high capacity of 1605 mAh g^−1^ at 100 mA g^−1^ was obtained. Moreover, Jin et al. coated BP‐graphene (BP‐G) particles with polyaniline (PANI) to prepare BP‐G/PANI composite.^[^ ^107^ ^]^ The BP‐G connected well with PANI through a covalent bond, and PANI ensured a stable SEI film and prevented the continuous accumulation of poor species (including Li fluorides and carbonates). Finally, the anode showed a high capacity of 910 mAh g^−1^ at 2.6 A g^−1^.

### 3.2 Phosphide‐Based Materials for LIBs

Owing to the high theoretical capacities, moderate electrical conductivity, and ultrahigh thermal stability, metal phosphides have been considered promising candidates as the anodes of LIBs.^[^ ^22^ ^]^ Currently, the emerged metal phosphides included Ni‐, Fe‐, Co‐, and Ge‐based phosphides, and most of them showed great potential in LIBs.

#### 3.2.1 Ni‐Based Phosphides

Ni‐based phosphides including NiP_2_,^[^ ^108^, ^109^ ^]^ NiP_3_,^[^ ^110^ ^]^ Ni_3_P,^[^ ^111^ ^]^ Ni_2_P,^[^ ^64^, ^65^ ^]^ and Ni_12_P_5_^[^ ^112^ ^]^ have been prepared and used in LIBs. Typically, Ni‐based phosphides can be divided into two categories: phosphorus‐rich and metal‐rich phases. Specifically, phosphorus‐rich phase, including NiP_2_^[^ ^108^, ^109^ ^]^ and NiP_3_,^[^ ^110^ ^]^ have relatively high theoretical capacity. For instance, Gillot et al. studied the Li^+^ storage mechanism of cubic and monoclinic NiP_2_ by directly growing NiP_2_ on Ni foam.^[^ ^108^ ^]^ It was established that during the first discharge process, cubic NiP_2_ reacted with Li^+^ through a conversion process, whereas the monoclinic NiP_2_ underwent an insertion process combined with a conversion reaction. Moreover, NiP_3_ was prepared and applied as the anode of LIBs, and after the complete discharge, the final product was confirmed to be Li_3_P.^[^ ^110^ ^]^ However, pure NiP_2_ and NiP_3_ have their own limitations, which fundamentally restricts the applications of Ni‐based phosphides for LIBs. To enhance their electrical conductivity and suppressed the volume changes, Lou et al. fabricated cubic NiP_2_ nanoparticles coated with carbon shells and then loaded them on CNTs (NiP_2_@C‐CNTs).^[^ ^109^ ^]^ Benefiting from the structural merits, a high cyclic stability (a capacity of 654 mAh g^–1^ after 1500 cycles at 5.0 A g^−1^) was achieved.

Different from the phosphorus‐rich phosphides mainly formed through the high temperature and/or hydrothermal reactions, metal‐rich phosphides are relatively simple, and they can be obtained under relatively low temperature and facile experimental procedures. Currently, the emerged metal‐rich phosphides included Ni_3_P,^[^ ^111^ ^]^ Ni_2_P,^[^ ^64^, ^65^ ^]^ and Ni_12_P_5_.^[^ ^112^ ^]^ For example, Zhang et al. prepared peapod‐like Ni_12_P_5_ nanoparticles coated on carbon fiber as the anode of LIBs.^[^ ^112^ ^]^ In comparison with bare Ni_12_P_5_, the composite anodes exhibited an enhanced capacity of 650 mAh g^−1^ after 200 cycles at 100 mA g^−1^. Moreover, Wu et al. embedded Ni_2_P nanoparticles within porous graphene networks generating a 3D yolk‐shell architecture.^[^ ^64^ ^]^ The open porous structure provides efficient channels for electron/ion transport, whereas the graphene networks supply enough space to accommodate the volume expansion of phosphides. Similarly, Wang et al. prepared carbon shell coated Ni_2_P sub‐microspheres using a facile solvothermal method,^[^ ^65^ ^]^ this distinctive core‐shell structure exhibited a high reversible capacity of 587 mAh g^−1^ after 400 cycles at 100 mA g^−1^.

#### 3.2.2 Fe‐Based Phosphides

Fe forms diverse metal phosphides, including FeP_2_,^[^ ^113^ ^]^ Fe_2_P,^[^ ^114^, ^115^ ^]^ and FeP.^[^ ^116^ ^]^ Particularly, amorphous Fe‐based phosphides buffered the volume changes, preventing performance degradation during cycles. For instance, Hall et al. synthesized amorphous FeP_2_ through a phosphorization approach.^[^ ^113^ ^]^ When used as the anode of LIBs, a reversible capacity of 906 mAh g^−1^ after 10 cycles at 137 mA g^−1^ was obtained. This moderate electrochemical result could be attributed to the low electrical conductivity and structural collapse.

**Figure 4:**
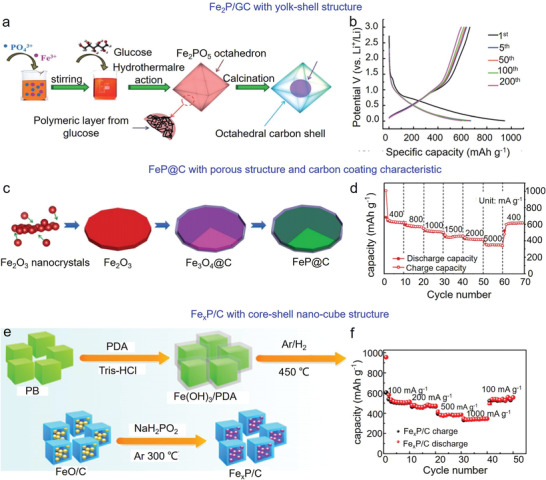
Fe‐based phosphide architectures used in LIBs. a) Schematic illustration of the synthesis of Fe_2_P/GC composite. b) Galvanostatic discharge–charge profiles at 0.1 A g^−1^. Reproduced with permission.^[^ ^114^ ^]^ Copyright 2016, The Royal Society of Chemistry. c) Schematic illustration of the preparation of FeP@C nanoplates. d) Rate capability of LIBs using FeP@C nanoplates as anode. Reproduced with permission.^[^ ^116^ ^]^ Copyright 2016, Royal Society of Chemistry. e) Schematic diagram of the preparation of Fe*_x_* P/C, and f) rate capability of LIBs using Fe*_x_* P/C anode. Reproduced with permission.^[^ ^117^ ^]^ Copyright 2018, Elsevier.

Hybridizing Fe‐based phosphides with carbonaceous materials can effectively improve the electrochemical results of LIBs. Yang et al. designed a yolk/shell architecture comprising of Fe_2_P particles wrapped in graphitized carbon (GC) (**Figure** **4**a).^[^ ^114^ ^]^ This structure resolved the issues including limited electron transfer and structural pulverization, displaying high discharge and charge capacities of 937 and 671 mAh g^−1^, respectively, corresponding to a CE of 71.6% (Figure 4b).

To enhance the electrochemical performance of LIBs further, it is necessary to design and fabricate Fe‐based phosphide/carbon hybrids with special structures. For instance, Han et al. synthesized porous FeP coated with carbon layers through a hydrothermal reaction followed by a phosphorization process (FeP@C) (Figure 4c).^[^ ^116^ ^]^ Benefiting from the porous structure and carbon coating characteristic, the as‐obtained anodes exhibited a high rate‐capability of 610 mAh g^−1^ at 400 mA g^−1^ (Figure 4d). Moreover, Zhang et al. synthesized a core‐shell nano‐cubic architecture comprising of Fe*_x_* P coated with carbon shells (Fe*_x_* P/C), and then employed it as the anodes of LIBs (Figure 4e).^[^ ^117^ ^]^ This structure possesses enough internal void/space to accommodate the volume expansion of Fe*_x_* P particles during Li^+^ insertion/extraction processes, whereas carbon shell improved the conductivity of anode and facilitated Li^+^ migration. Eventually, Fe*_x_* P/C anodes delivered a capacity of 665 mAh g^−1^ after 200 cycles at 0.1 A g^−1^, and significant rate capability with a capacity of 346 mAh g^−1^ at 1.0 A g^−1^ (Figure 4f).

#### 3.2.3 Co‐Based Phosphides

**Figure 5:**
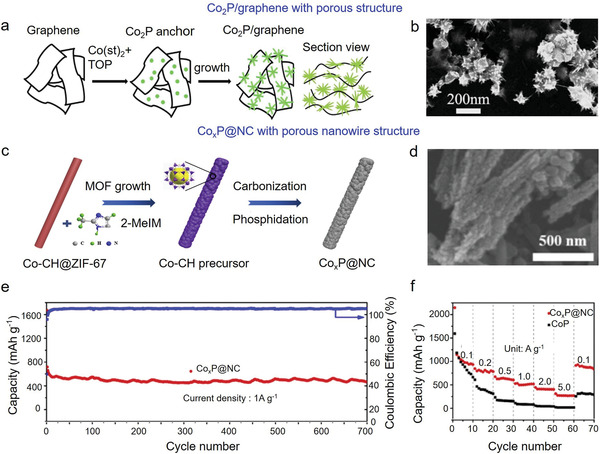
Co‐based phosphides used in LIBs. a) Schematic illustration of the preparation of Co_2_P/graphene composite, and b) the typical structure. Reproduced with permission.^[^ ^118^ ^]^ Copyright 2015, Elsevier. c) Schematic illustration of the synthesis of Co*_x_* P@NC nanowires, and d) the typical morphology. e) Long‐term cyclic performance of Co*_x_* P@NC at 1.0 A g^−1^, and f) rate capability of Co*_x_* P@NC and CoP. Reproduced with permission.^[^ ^119^ ^]^ Copyright 2020, Elsevier.

Co‐based phosphides possess various types, such as Co_2_P,^[^ ^118^ ^]^ Co*_x_* P,^[^ ^119^ ^]^ CoP,^[^ ^120^ ^]^ and CoP_3_.^[^ ^121^ ^]^ Bare Co‐based phosphides are merely used in LIBs, because of the harsh synthesis conditions, low electrical conductivity, and apparent volume expansion, meanwhile, Co‐based phosphides combined with highly conductive carbonaceous materials and polymers were confirmed to be suitable candidates. For instance, Lu et al. prepared Co_2_P/graphene hybrid using a one‐pot solution method (**Figure** **5**a,b).^[^ ^118^ ^]^ The as‐prepared Co_2_P/graphene anodes exhibited better electrochemical performance as compared with those of their two counterparts, attributing to the effective combination between Co_2_P and graphene sheets, high electron transfer speed, and well dispersion of Co_2_P nanoparticles on graphene sheets. Recently, Liu et al. encapsulated Co*_x_* P particles on tube‐sheath nanowires using MOFs as precursor, and formed Co*_x_* P@N‐doped carbon (NC) composite (Figure 5c,d).^[^ ^119^ ^]^ Co*_x_* P@NC anodes delivered a high capacity of 526 mAh g^−1^ after 700 cycles at 1.0 A g^−1^ (Figure 5e,f). Similarly, CoP/heteroatom‐doped carbon was also confirmed to exhibit good electrochemical results.^[^ ^120^ ^]^

In addition to carbonaceous materials, polymers were also emerged and employed as conductive additives. For instance, Liu et al. prepared polypyrrole (PPy)‐coated micro‐cubic CoP_3_ (CoP_3_@PPy) composite using a coprecipitation method.^[^ ^121^ ^]^ This structure alleviated well the volume expansion and promoted fast electron transfer, thereby, a high discharge capacity of 1615 mAh g^−1^ at 100 mA g^−1^, and good cyclic stability of 650 mAh g^−1^ after 200 cycles at 500 mA g^−1^ were achieved.

#### 3.2.4 Other Phosphides

Besides the abovementioned metal phosphides, other phosphides have been also prepared and utilized. Currently, the emerged phosphides include GeP_3_,^[^ ^122^ ^]^ GeP,^[^ ^123^ ^]^ GeP_5_,^[^ ^76^ ^]^ V_4_P_7_,^[^ ^71^ ^]^ SiP_2_,^[^ ^78^ ^]^ GaP,^[^ ^124^ ^]^ MoP,^[^ ^125^ ^]^ and Sn_4_P_3_.^[^ ^126^ ^]^ For instance, Li et al. prepared GeP_5_/carbon composite via a high‐energy ball milling method.^[^ ^77^ ^]^ It was revealed that GeP_5_/carbon anodes exhibited a remarkable reversible capacity of 2300 mAh g^−1^, and a high initial CE of ≈95%. Moreover, Fu et al. constructed a hybrid comprising of MoP@N‐doped CNFs (MoP@NCNFs) through the electrospinning approach.^[^ ^125^ ^]^ MoP nanoparticle was uniformly distributed within CNFs, effectively reducing the pulverization and collapse of active materials during cycles. Correspondingly, electrochemical results showed a capacity of 377 mAh g^−1^ after 1300 cycles at 2.0 A g^−1^.

## 4 Phosphorus‐ and Phosphide‐Based Materials for SIBs

SIBs have been considered a promising candidate for replacing the conventional LIBs, owing to the reserve abundance of sodium in the earth and low costs. However, the slow reaction kinetics originating from large ion radius hindered their commercialization. Designing and fabricating of phosphorus‐ and phosphide‐based materials with high theoretical capacity and stable structural integrity can resolve the above issues, and thereby promoting their practical utilizations. In the following parts, SIBs using phosphorus‐ and phosphide‐based materials will be discussed (**Table** **2**).

**Table 2:** Phosphorus‐ and phosphide‐based materials for SIBs

Materials	Morphology	Synthesis method	Rate performance	Cyclic performance	Ref.
C@P/GA	3D porous graphene structure	Vapor‐redistribution	878 mAh g^−1^ at 5.2 A g^−1^	1095 mAh g^−1^ after 200 cycles at 2.6 A g^−1^	[143]
P@N‐MPC	Porous polyhedral structure	Vaporization–condensation–conversion	291 mAh g^−1^ at 8.0 A g^−1^	450 mAh g^−1^ after 1000 cycles at 1.0 A g^−1^	[147]
P@NCNFs	Porous nanofiber structure	Ball‐milling and electrospinning	343 mAh g^−1^ at 10.0 A g^−1^	619 mAh g^−1^ after 1000 cycles at 2.0 A g^−1^	[150]
Sn_4_P_3_‐GA	Hierarchical porous structure	Rapid low‐temperature phosphidation	403 mAh g^−1^ at 2.0 A g^−1^	657 mAh g^−1^ after 100 cycles at 0.1 A g^–1^	[167]
Sn_4_P_3_@CNFs	Porous nanofiber structure	Electrospinning and thermal treatment	321 mAh g^−1^ at 5.0 A g^−1^	336 mAh g^−1^ after 500 cycles at 1.0 A g^–1^	[168]
FeP@NPC	Nanofiber structure	Electrospinning and phosphidation	250 mAh g^−1^ at 5.0 A g^–1^	253 mAh g^−1^ after 300 cycles at 1.0 A g^–1^	[171]
H‐FeP@C@GR	Porous spherical structure	Hydrothermal reaction and phosphidation	237 mAh g^−1^ at 1.6 A g^–1^	400 mAh g^−1^ after 250 cycles at 0.1 A g^–1^	[176]
CoP@C‐rGO‐Ni foam	Core/shell polyhedral structure	Low‐temperature phosphidation	155 mAh g^−1^ at 1.6 A g^−1^	473 mAh g^−1^ after 100 cycles at 0.1 A g^−1^	[180]
Co_2_P/Sn@NC	Plum Pudding‐Like structure	Thermally treated	147 mAh g^−1^ at 10. A g^−1^	168 mAh g^−1^ after 10000 cycles at 1.0 A g^−1^	[184]
Ni_2_P@C/GA	Core/shell structure	Solvothermal reaction and phosphidation	122 mAh g^−1^ at 2.0 A g^−1^	124 mAh g^−1^ after 2000 cycles at 1.0 A g^−1^	[193]

### 4.1 Phosphorus‐Based Materials for SIBs

#### 4.1.1 Pure Phosphorus

Similar to that of LIBs, pure RP in SIBs suffered from various crucial issues including serious volume expansion, low electrical conductivity, and slow reaction kinetics,^[^ ^127^ ^]^ leading to a rapid capacity decrease. To enhance the electrochemical result of phosphorus, special structures were elaborately designed and constructed. For instance, Liu et al. prepared RP through the redox reactions, and then converted it into iodine‐doped hollow nanoporous RP (HNPRP).^[^ ^127^ ^]^ Benefiting from the effective alleviation of volume expansion and reduced ion transport length, the as‐prepared anodes exhibited a reversible capacity of 1658 mAh g^−1^ after 100 cycles at 260 mA g^−1^, and great long‐life cyclic stability with a reversible capacity of 857 mAh g^−1^ after 1000 cycles at 2.6 A g^−1^. Similarly, Santhoshkumar et al. synthesized multichannel RP nanoporous (MRPN) architectures through a solvothermal method.^[^ ^128^ ^]^ This structure provided efficient electron/ion transport networks and buffered the volume expansion of RP particles, exhibiting a high capacity of 1814 mAh g^−1^, and a reversible capacity of 735 mAh g^−1^ after 400 cycles at 3.2 A g^−1^.

#### 4.1.2 Phosphorus/Carbon Hybrids

##### Phosphorus/Carbon Black

Combining phosphorus with carbonaceous materials can overcome the obstacles originated from low electronic conductivity and structural pulverization. The simplest strategy is to mix phosphorus with carbon black, and the efficient strategy included high‐energy ball milling method.^[^ ^129^, ^130^ ^]^ For instance, Qian et al. fabricated RP/carbon black through ball milling method, and a high capacity of 1764 mAh g^−1^ after 100 cycles was achieved in SIBs.^[^ ^129^ ^]^ By controlling the temperature and pressure conditions, amorphous RP was converted into orthorhombic BP with reduced particle size. For instance, Peng et al. synthesized BP‐carbon composite using RP and super P as raw materials.^[^ ^131^ ^]^ The nano‐sized BP distributed uniformly within carbon networks, and the volume expansion of BP was efficiently alleviated. As a result, BP‐carbon anodes exhibited a capacity of 1381 mAh g^−1^ after 100 cycles at 100 mA g^−1^, corresponding to a capacity retention of 90.5%.

##### Phosphorus/CNTs

Owing to the high length‐to‐width ratio, CNTs can form an interconnected network with a long‐range conductivity,^[^ ^22^ ^]^ thereby facilitating the utilizations of phosphorus in SIBs.^[^ ^132^, ^133^, ^134^, ^135^ ^]^ Li et al. fabricated P/CNTs through the manual grinding of commercial RP and CNTs.^[^ ^132^ ^]^ The as‐prepared anodes delivered a high initial capacity of 1675 mAh g^−1^ at 143 mA g^−1^, and a capacity retention of 76.6% after 10 cycles. Through designing and constructing unique nanostructures of anodes, Yu et al. synthesized P@YP (microporous carbon) and P@CNT composite by using the vaporization‐deposition‐conversion method.^[^ ^133^ ^]^ This structure alleviated large volume changes and shortened ion diffusion length during cycles, and a stable SEI film was generated to ensure the good long‐term cyclic stability.

##### Phosphorus/Graphene

Graphene sheets can be employed to hybridize with phosphorus to enhance fast electron transfer, electrochemical reaction kinetics, and structural integrity. For instance, Pei et al. prepared RP/graphene scrolls (P—G) and RP/planar graphene sheet (P/G) composites, and utilized them as the anodes of SIBs.^[^ ^136^ ^]^ Benefiting from the structural merits, good electrochemical results were simultaneously achieved. Moreover, ball milling and vaporization‐condensation methods were developed to synthesize various graphene/phosphorus architectures.^[^ ^137^, ^138^, ^139^, ^140^, ^141^ ^]^ However, in the conventional vaporization‐condensation process, RP inevitably produces WP residue, inducing the safety issues, and thereby limiting its practical application. Thereafter, promising solutions have been proposed and utilized to solve this issue. For instance, Zhou et al. verified that the generation of WP can be significantly inhibited using sulfur‐doped phosphorus strategy.^[^ ^142^ ^]^ A hybrid comprising of sulfur‐doped RP loaded on rGO (S‐P/rGO) was fabricated, and S‐P/rGO anode showed a high capacity of 2313 mAh g^−1^ and significant rate capability with a capacity of 293 mAh g^−1^ at 30 C.

Graphene aerogel (GA) is an intriguing architecture with abundant active sites, large surface area, and high pore volume, thereby promoting the electron/ion transfer and allowed a high phosphorus content. For instance, Gao et al. distributed RP particles evenly within a PPy‐covered GA matrix (C@GA), achieving a 3D porous C@P/GA anode with a high reversible capacity of 1095 mAh g^−1^ at 2.6 A g^−1^.^[^ ^143^ ^]^ Similarly, Yan et al. successfully encapsulated crystalline RP (CRP) nanorods within 3D rGO aerogel (3D rGA).^[^ ^144^ ^]^ Benefiting from the strong interactions between CRP and rGA, CRP‐rGA anode delivered high specific capacities of 918 and 860 mAh g^−1^ at 1.0 A g^−1^ and 2.0 A g^−1^, respectively, after 30 cycles.

##### Phosphorus/Porous Carbon Matrices

**Figure 6:**
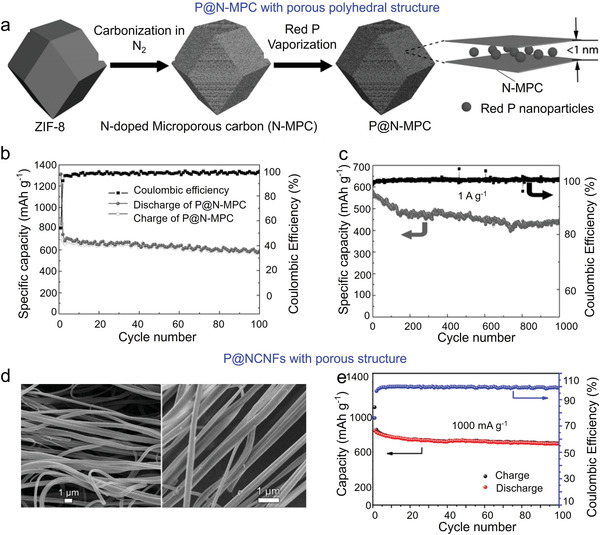
Phosphorus‐based materials used in SIBs. a) Schematic illustration of the preparation of P@N‐MPC. Cyclic performance of P@N‐MPC at b) 0.15 and c) 1.0 A g^−1^. Reproduced with permission.^[^ ^147^ ^]^ Copyright 2017, Wiley‐VCH. d) SEM images of P@NCNFs nanofibers. e) Cyclic stability of P@NCNFs at 1000 mA g^−1^. Reproduced with permission.^[^ ^150^ ^]^ Copyright 2017, Elsevier.

In SIBs, porous carbon with abundant pores and large surface areas also shortened the electron/ion diffusion paths, improved phosphorus content, and restricted the volume changes. The most extensively employed method is vaporization‐condensation strategy.^[^ ^145^, ^146^, ^147^, ^148^, ^149^ ^]^ For instance, Li et al. confined nanoscale amorphous RP into N‐doped microporous carbon matrix (P@N‐MPC) derived from ZIF‐8 (**Figure** **6**a).^[^ ^147^ ^]^ The structure established a highly conductive path and promoted electron/ion diffusion; RP restricted within nanoscale chamber alleviated the volume change during cycles; the porous structure ensured efficient contact of the electrolyte with RP. Owing to the above merits, P@N‐MPC anodes showed a high reversible capacity of 600 mAh g^−1^ at 150 mA g^−1^, and a capacity of 450 mAh g^−1^ after 1000 cycles at 1.0 A g^−1^ (Figure 6b,c).

Recently, novel synthesis approaches have been emerged and utilized to prepare phosphorus‐based materials. For instance, Liu et al. embedded phosphorus nanoparticles within porous N‐doped CNFs (P@NCNFs) using the electrospinning method.^[^ ^150^ ^]^ As shown in Figure 6d, the amorphous phosphorus nanoparticles distributed well within carbon matrix, and this structure promoted the fast transport of electrons/ions, and reduced the collapse of active materials induced by volume changes. Therefore, the as‐prepared P@NCNFs anode exhibited a long cyclic stability with a capacity of 698 mAh g^−1^ at 1000 mA g^−1^, accompanying with a capacity retention of 82.3% (Figure 6e).

#### 4.1.3 Phosphorus/Conductive Polymers

Combining phosphorus with polymers can also achieve the high Na^+^ storage performance.^[^ ^151^, ^152^, ^153^ ^]^ For instance, Zhang et al. precipitated poly (3,4‐ethylenedioxythiophene) (PEDOT) nanofibers on exfoliated few‐layer BP (E‐BP) nanosheets.^[^ ^151^ ^]^ Owing to the structural merits, the charge‐transfer kinetics was enhanced, realizing good contact with the electrolyte. Finally, the as‐prepared anodes exhibited a high reversible capacity of 1600 mAh g^−1^ at 100 mA g^−1^, and the capacity maintained at 1078 mAh g^−1^ after 100 cycles.

### 4.2 Phosphide‐Based Materials for SIBs

Phosphide‐based materials with high theoretical capacity and energy density can be adopted as the anodes of SIBs, but they have various disadvantages similar to that in LIBs. To solve these issues, diverse strategies have been explored and employed, and several metal phosphides including Sn‐, Fe‐, and Co‐based phosphides, have been verified to exhibit good electrochemical performance in SIBs.

#### 4.2.1 Sn‐Based Phosphides

Tin phosphides, such as Sn_4_P_3_^[^ ^154^ ^]^ SnP_3_,^[^ ^155^ ^]^ and SnP,^[^ ^156^ ^]^ have high theoretical capacity and excellent electronic conductivity. Particularly, Sn_4_P_3_ with a layered structure has been widely studied as an anode for SIBs. For example, Li et al. prepared tin phosphide and amorphous Sn‐P (Sn_4+_ *_x_* P_3_@(Sn‐P)) composite using the ball milling method,^[^ ^157^ ^]^ and Liu et al. prepared Sn_4_P_3_ nanoparticles through the solvothermal method.^[^ ^80^ ^]^ When used as the anodes of SIBs, they both exhibited good electrochemical results in terms of specific capacity and rate capability. However, Sn_4_P_3_ normally suffered from the tin aggregation and volume expansion during electrochemical reactions, contributing to the obvious degradation of electrochemical performance.^[^ ^158^, ^159^ ^]^

**Figure 7:**
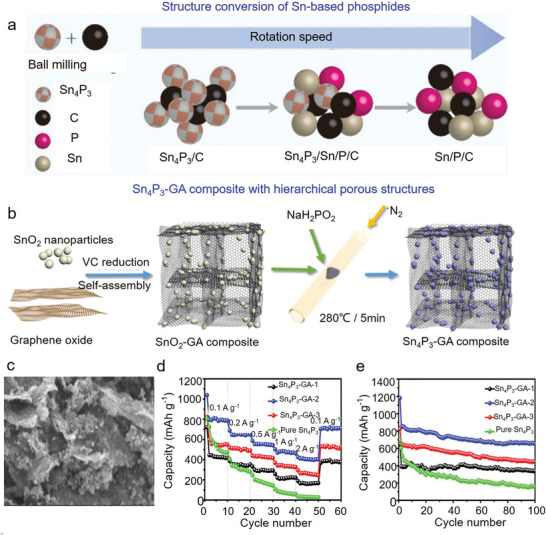
Sn‐based phosphides used in SIBs. a) Schematic illustration of the tuning of Sn‐based structures. Reproduced with permission.^[^ ^160^ ^]^ Copyright 2019, Elsevier. b) Schematic illustration of the synthesis of Sn_4_P_3_‐GA composite, and c) the typical morphology. d) Rate capability and e) cyclic stability (0.1 A g^−1^) of Sn_4_P_3_‐GA composites. Reproduced with permission.^[^ ^167^ ^]^ Copyright 2018, Elsevier.

To address these above issues, hybridizing Sn_4_P_3_ with carbonaceous materials should be the promising strategy, meanwhile, carbonaceous materials can improve the electronic conductivity, thus promoting fast electron transfer of SIBs. Ball milling method is the most extensively utilized approach for fabricating Sn_4_P_3_/carbon architectures.^[^ ^160^, ^161^ ^]^ As a representative work, Saddique et al. fabricated (Sn_4_P_3_/carbon), ternary (Sn/P/carbon), and quaternary (Sn_4_P_3_/Sn/P/carbon) composites through the ball milling method (**Figure** **7**a).^[^ ^160^ ^]^ They revealed that Sn_4_P_3_/Sn/P/C anodes showed the best electrochemical performance, and a high reversible capacity of 382 mAh g^−1^ after 300 cycles at 500 mA g^−1^ was achieved, corresponding to a capacity retention of 86%. Similarly, Mulder et al. prepared Sn_4_P_3_‐P/graphene composite via the ball milling method.^[^ ^161^ ^]^ Electrochemical testing revealed high‐rate capacity retentions of >550 and 371 mAh g^−1^ at 1.0 and 2.0 A g^−1^, respectively, over 1000 cycles.

Although ball milling method can produce Sn_4_P_3_/carbon, however, it is hard to achieve the ideal morphology/structure with high purity. Nevertheless, various strategies have been recently explored and utilized to prepare Sn_4_P_3_/carbon with intriguing micro‐and nanostructures. Of them, the direct phosphorization of Sn‐based precursor should be the most efficient approach, besides PH_3_ with noxious property,^[^ ^162^, ^163^ ^]^ RP was the widely adopted phosphorus source.^[^ ^164^, ^165^, ^166^ ^]^ For instance, Fan et al. synthesized Sn_4_P_3_@carbon spheres using the aerosol spray‐pyrolysis and phosphorization method.^[^ ^164^ ^]^ Electrochemical results revealed a high capacity of 800 mAh g^−1^, accompanying with a capacity decay of 0.09% per cycle. To further alleviate the volume expansion of Sn_4_P_3_, yolk‐shelled structure was designed and constructed. Typically, Yu et al. prepared uniform yolk‐shell Sn_4_P_3_@carbon nanospheres for high‐performance SIBs.^[^ ^165^ ^]^ Benefiting from the structural merits, SIBs delivered a high capacity of 421 mAh g^−1^ at 3.0 C, and stable cyclic performance with a capacity of 360 mAh g^−1^ after 400 cycles at 1.5 C. Similar electrochemical results were also achieved by using yolk‐shelled Sn_4_P_3_@carbon nanobox as the anodes of SIBs.^[^ ^166^ ^]^

Other carbonaceous materials, including graphene,^[^ ^167^ ^]^ and CNFs,^[^ ^168^ ^]^ were also involved in fabricating Sn_4_P_3_/carbon architectures. As a representative work, Pan et al. prepared Sn_4_P_3_‐GA composite using a rapid low‐temperature phosphorization process (Figure 7b).^[^ ^167^ ^]^ Figure 7c shows the SEM images of Sn_4_P_3_‐GA composite. This structure reduced the size of Sn_4_P_3_ and provided a 3D network, lowering the tension induced by volume expansion and enhancing the electronic conductivity. As a result, the as‐prepared Sn_4_P_3_‐GA anodes showed a reversible capacity of 657 mAh g^−1^ at 0.1 A g^−1^, and a good rate capability with a capacity of 403 mAh g^−1^ at 2.0 A g^−1^ (Figure 7d,e). Moreover, Ran et al. encapsulated Sn_4_P_3_ into porous and free‐standing CNFs using an electrospinning method combined with the heat treatment process (Sn_4_P_3_@CNFs).^[^ ^168^ ^]^ The 3D conductive network facilitated the electron/ion transport, whereas the void and space within CNFs alleviated the volume changes during cycles.

#### 4.2.2 Fe‐Based Phosphides

**Figure 8:**
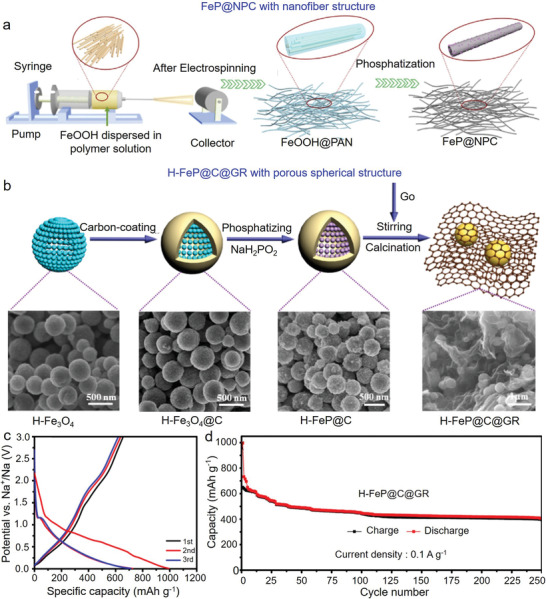
Fe‐based phosphides used in SIBs. a) Schematic illustration of the synthesis of FeP@NPC nanofiber. Reproduced with permission.^[^ ^171^ ^]^ Copyright 2020, Elsevier. b) Schematic illustration of the synthesis of H‐FeP@C@GR composite. c) Discharge/charge profiles of the initial three cycles and d) long‐term cyclic performance of H‐FeP@C@GR at 0.1 A g^−1^. Reproduced with permission.^[^ ^176^ ^]^ Copyright 2017, American Chemical Society.

Fe‐based phosphides recently emerged as the anodes of SIBs. Of them, FeP is the most commonly examined material in SIBs, with a high theoretical capacity of 924 mAh g^−1^. However, similar to other phosphides, FeP has poor conductivity and large volume expansion during cycles. To resolve these issues, nanostructured FeP has been designed and hybridized with carbonaceous materials. To date, the emerged FeP structures included nanoparticles,^[^ ^169^, ^170^, ^171^, ^172^ ^]^ nanorods,^[^ ^173^, ^174^ ^]^ and nanospheres.^[^ ^175^, ^176^ ^]^ For instance, Lim et al. prepared porous FeP/carbon composite through low‐temperature phosphorization of MOFs.^[^ ^72^ ^]^ FeP nanoparticles distributed uniformly within carbon scaffold, realizing the rapid electron/ion transport, alleviating volume changes, and providing abundant active sites for electrochemical reactions. Similarly, Shi et al. prepared FeP nanoparticles wrapped in 3D interconnected N/P co‐doped carbon fibers using the electrospinning method (**Figure** **8**a).^[^ ^171^ ^]^ Such a design concept prevented the agglomeration of FeP, and thereby FeP@NPC exhibited a reversible capacity of 253 mAh g^−1^ after 300 cycles at 1.0 A g^−1^. To enhance the utilization of FeP further, Li et al. synthesized a copper and carbon‐combined yolk‐shelled FeP (YS‐Cu‐FeP@C).^[^ ^175^ ^]^ The doped N atoms increased the electronic conductivity, and copper increased the electronic conductivity and promoted the migration of Na^+^. Similarly, Wang et al. synthesized graphene (GR) encapsulated hollow FeP@carbon composite (H‐FeP@C@GR) for SIBs (Figure 8b‐c).^[^ ^176^ ^]^ The thin carbon layer wrapped by hollow FeP (H‐FeP) nanospheres formed a 3D structure, accommodating the volume expansion during cycles and providing a high‐speed way for electron/ion transfer. Electrochemical testing revealed a high capacity of 400 mAh g^−1^ after 250 cycles at 100 mA g^−1^ (Figure 8d).

#### 4.2.3 Co‐Based Phosphides

Although Co is not active upon sodiation, Co‐based phosphides can react electrochemically with Na^+^. Particularly, CoP is the most commonly used anode material in SIBs. Initially, Li et al. prepared CoP particles using a simple ball milling method, and electrochemical results revealed a high reversible capacity of 770 mAh g^−1^.^[^ ^177^ ^]^ However, the electrochemical performance of CoP anodes prepared using ball milling method decreased rapidly during cycles, and the particle size became larger and uneven, resulting in poor rate capability. To resolve this issue, Zhang et al. prepared CoP nanoparticle uniformly embedded within N‐doped CNSs through the reactions between cobalt‐based MOFs and RP.^[^ ^178^ ^]^ The as‐achieved CoP/CNSs anodes delivered a capacity of 174 mAh g^−1^ at 20 A g^−1^, and a capacity of 386 mAh g^−1^ after 900 cycles at 1.0 A g^−1^. Similarly, Chang et al. synthesized S‐doped CoP (S‐CoP) nanoparticle loaded on hollow N‐doped porous carbon (NPC) using the low‐temperature phosphorization and sulfurization treatment method.^[^ ^179^ ^]^ The synergistic effects between S and N doping provided abundant active sites, and improved the overall conductivity; thus, a discharge capacity of 230 mAh g^−1^ after 370 cycles at 200 mA g^−1^ was obtained, accompanying with a capacity retention of 91%.

To further enhance the electrochemical performance of Co‐based phosphides from the aspects of improving the electrical conductivity and suppressing the volume expansion of active component, Ge et al. prepared CoP@carbon polyhedrons on 3D rGO network through a low‐temperature phosphorization process.^[^ ^180^ ^]^ The core/shell structure provided enough space to alleviate volume change during cycles, carbon shell prevented the decomposition and aggregation of CoP particles, and 3D rGO/Ni foam improved the electronic conductivity significantly. Moreover, Zhang et al. prepared CoP nanowires on carbon paper (CP) and then introduced conductive polymer PPy as a protective layer.^[^ ^181^ ^]^ This structure buffered the volume expansion and enhanced charge transfer, contributing to a stable cyclic performance and high rate capability. Furthermore, CoP combined with other metal phosphides can be also used to improve the Na^+^ storage performance,^[^ ^182^, ^183^ ^]^ indicating their potential applications in SIBs.

Other cobalt phosphides, such as Co_2_P,^[^ ^184^, ^185^, ^186^ ^]^ CoP_3_,^[^ ^187^ ^]^ and CoP_4_^[^ ^188^ ^]^ have also been reported as the anodes of SIBs. For example, Ren et al. prepared Co_2_P/Sn@N‐doped carbon nanobox comprising of hollow CoSn(OH)_6_ nanocubes coated with carbon layers.^[^ ^184^ ^]^ The as‐achieved anode exhibited a high reversible capacity of 394 mAh g^−1^ at 100 mA g^−1^.

#### 4.2.4 Other Phosphides

Other metal phosphides such as Zn_3_P_2_,^[^ ^79^ ^]^ GeP,^[^ ^189^ ^]^ Se_4_P_4_,^[^ ^190^ ^]^ Mo*_x_* P,^[^ ^70^, ^191^ ^]^ Cu*_x_* P,^[^ ^63^, ^192^ ^]^ and Ni*_x_* P,^[^ ^193^, ^194^, ^195^ ^]^ have been explored and employed as the anode materials of SIBs. For instance, Miao et al. synthesized a 3D interconnected porous structure comprising Ni_2_P@carbon/GA (Ni_2_P@C/GA) through the solvothermal method followed by phosphorization.^[^ ^193^ ^]^ This structure buffered the volume change of Ni_2_P, and prevented the agglomeration and pulverization of Ni_2_P during cycles; thus, a specific capacity of 124 mAh g^−1^ after 2000 cycles at 1.0 A g^−1^ was finally achieved.

## 5 Phosphorus‐ and Phosphide‐Based Materials for PIBs

Similar to those of LIBs and SIBs, phosphorus‐ and phosphide‐based materials have also attracted tremendous attention in PIBs (**Table** **3**). Additionally, they experienced almost similar issues to those in LIBs and SIBs, including volume expansion and slow reaction kinetics. Therefore, the strategies proposed to solve these issues are similar.

**Table 3:** Phosphorus‐ and phosphide‐based materials for PIBs

Materials	Morphology	Synthesis method	Rate performance	Cyclic performance	Ref.
P@TBMC	Mesoporous structure	Vaporization–condensation–conversion	136 mAh g^−1^ at 2.0 A g^−1^	244 mAh g^−1^ after 200 cycles at 0.5 A g^−1^	[196]
P@N‐PHCNFs	Porous hollow structure	Electrospinning method	342 mAh g^−1^ at 5.0 A g^−1^	465 mAh g^−1^ after 800 cycles at 2.0 A g^−1^	[204]
P@AC@PPy	Porous structure	Vaporization–deposition–conversion	132 mAh g^−1^ at 0.5 A g^−1^	220 mAh g^−1^ after 200 cycles at 0.05 A g^−1^	[206]
SnP@carbon	Porous spherical structure	Hydrothermal and thermal treatment	257 mAh g^−1^ at 1.0 A g^−1^	236 mAh g^−1^ after 200 cycles at 1.0 A g^−1^	[208]
SnP_0.94_@graphene oxide	Nanoplate structure	Hot‐injection method	57 mAh g^−1^ at 1.0 A g^−1^	106 mAh g^−1^ after 100 cycles at 0.2 A g^−1^	[210]
AC@CoP/NCNTs/CNFs	Porous core/shell structure	Electrospinning and phosphidation	292 mAh g^−1^ at 3.2 A g^−1^	250 mAh g^−1^ after 1000 cycles at 0.2 A g^−1^	[213]
Co_2_P@rGO	Ultrafine nanorod structure	Colloidal mesostructured method	141 mAh g^−1^ at 2.0 A g^−1^	374 mAh g^−1^ after 50 cycles at 0.02 A g^−1^	[214]
MoP@PC	Porous nanodot structure	Mixing and annealing treatment	208 mAh g^–1^ at 5.0 A g^–1^	240 mAh g^−1^ after 1000 cycles at 1.0 A g^−1^	[215]
MoP@N/P‐co‐CNTs	Hollow nanotube structure	Solvothermal method and phosphidation	275 mAh g^–1^ at 1.0 A g^–1^	255 mAh g^−1^ after 120 cycles at 1.0 A g^−1^	[216]
Se_3_P_4_@mesoporous carbon	Hierarchical structure	Situ combination reaction	498 mAh g^–1^ at 1.0 A g^–1^	400 mAh g^−1^ after 300 cycles at 1.0 A g^−1^	[217]

### 5.1 Phosphorus/Carbon Hybrids

#### 5.1.1 Phosphorus/Graphite

As discussed in LIBs, graphite can form P—C and P—O—C bonds with phosphorus, which combines phosphorus and graphite tightly, improving the entire structural integrity. For instance, Wu et al. prepared RP/graphite using ball milling method.^[^ ^56^ ^]^ Amorphous RP was ground into small size and formed P—C bond with graphite, this design not only alleviated the volume expansion, but also shortened the transport of K^+^. Similarly, Sultana et al. prepared BP‐C (graphite) composite.^[^ ^57^ ^]^ Owing to the high content of BP in BP—C composite, a capacity of 617 mAh g^−1^ was achieved. In addition, Jin et al. further examined the in‐depth working mechanism of BP in PIBs,^[^ ^58^ ^]^ and they established that the final potassiation product was K_3_P, which could degrade the electrochemical performance, because of the high formation energy and large ion diffusion coefficient.

#### 5.1.2 Phosphorus/CNTs

CNTs were also investigated as the conductive additive of phosphorus‐based anodes for PIBs. For instance, Liu et al. encapsulated RP into CNT‐backboned mesoporous carbon (TBMC) using the vaporization‐condensation‐conversion method.^[^ ^196^ ^]^ This structure enabled fast electron transfer and provided sufficient void/space to accommodate the volume expansion of RP. Similarly, Chang et al. prepared RP/multi‐walled CNT (MWCNT)‐Ketjen black (KB),^[^ ^197^ ^]^ and it was revealed that the formation of P‐C bond limited the alloying process between K^+^ and RP, resulting in a low K^+^ storage capability.

Owing to the different functions of P—C bonds in PIBs and LIBs/SIBs, the effects of P—C bonds on final K^+^ storage behavior should be systematically investigated.^[^ ^198^, ^199^ ^]^ As a representative work, Peng et al. prepared RP/carboxylic group CNT (P/CGCNT) composite through the simple ball milling method, and they confirmed that the generation of P—C bonds was responsible for the high K^+^ storage behavior.^[^ ^198^ ^]^ Therefore, the as‐prepared P/CGCNT anodes exhibited a high initial capacity of 589 mAh g^−1^ at 100 mA g^−1^, and then maintained at 402 mAh g^−1^ after 110 cycles, corresponding to a capacity retention of 68%.

#### 5.1.3 Phosphorus/Graphene

Graphene can be hybridized with phosphorus in PIBs. As a representative work, Wang et al. prepared RP nanoparticles wrapped within rGO matrix using the solvothermal method.^[^ ^42^ ^]^ rGO increased the structural disorder, reduced the energy barrier of chemical reactions, and generated the P—C bonds; thereby, a capacity of 253 mAh g^−1^ after 500 cycles at 500 mA g^−1^ was achieved. Moreover, Muhammad et al. synthesized a 3D porous RP‐graphdiyne (3D‐PGDY) composite as the anodes of PIBs.^[^ ^200^ ^]^ This structure provided abundant active sites and sufficient space, contributing to a high K^+^ storage capacity of 1064 mAh g^−1^ and small volume change (2.43%) during cycles.

#### 5.1.4 Phosphorus/Porous Carbon Matrices

Porous carbon materials should be the ideal candidate for hosting pure phosphorus, and the most widely used strategy is vaporization–condensation method.^[^ ^201^, ^202^, ^203^ ^]^ For instance, Xiao et al. encapsulated RP nanoparticles into commercial porous carbon.^[^ ^203^ ^]^ With a high RP content of 59.4 wt.%, the as‐prepared anodes exhibited a high capacity of 744 mAh g^−1^ at 0.1 A g^−1^, and a reversible capacity of 212 mAh g^−1^ after 1000 cycles at 3.2 A g^−1^. Recently, various novel approaches have been emerged and used to prepare RP/carbon composites, and significantly enhanced electrochemical results were achieved.^[^ ^204^, ^205^ ^]^ Typically, Wu et al. encapsulated RP nanoparticles within N‐doped porous/hollow CNFs (N‐PHCNFs) using electrospinning method.^[^ ^204^ ^]^ This structure has abundant P—C chemical bonds and N‐doped active sites, alleviating the volume expansion during cycles and enhancing the entire structural stability. Experimental results indicated a different alloying mechanism of RP and confirmed the formation of K_4_P_3_ interphase. When used as the anode, a high capacity of 465 mAh g^−1^ after 800 cycles at 2.0 A g^−1^ was observed. Moreover, RP nanoparticles loaded onto biomass‐derived carbon matrix were also confirmed to have a high K^+^ storage performance.^[^ ^205^ ^]^

#### 5.1.5 Phosphorus/Conductive Polymers

Additionally, phosphorus combined with conductive polymers can be used as the anodes of PIBs. For example, Fang et al. synthesized P@active carbon@PPy composite using the vaporization‐deposition‐conversion method.^[^ ^206^ ^]^ The PPy layer enhanced the antioxidant capacity and avoided the direct contact between active material and the electrolyte as well as reduced the side reactions. Consequently, the as‐synthesized P@AC@PPy anodes exhibited a capacity of 220 mAh g^−1^ after 200 cycles at 50 mA g^−1^.

### 5.2 Phosphide‐Based Materials for PIBs

Phosphides usually exhibit metallic properties and excellent thermal stability, and the mainly emerged phosphides for PIBs included Sn‐, Fe‐, Co‐ and Mo‐based phosphides. In the following sections, these phosphides are discussed in detail.

#### 5.2.1 Sn‐Based Phosphides

Sn_4_P_3_ has been investigated as the anode of PIBs, but the achieved electrochemical results still cannot meet the demands for practical applications.^[^ ^207^ ^]^ Hybridizing phosphides with carbonaceous materials should be a simple and efficient solution to cater to the utilizations in PIBs.^[^ ^73^, ^74^, ^75^ ^]^ For instance, Zhang et al. synthesized Sn_4_P_3_/carbon black through the ball milling method.^[^ ^73^ ^]^ It was revealed that K‐Sn (K_4_Sn_23_, KSn) and K—P (K_3−_ *_x_* P) alloys were formed upon discharging, alleviating the volume change of phosphides. Consequently, a high discharge capacity of 588 mAh g^−1^ at 50 mA g^−1^ was achieved, and the capacity gradually stabilized at 307 mAh g^−1^ after 50 cycles. Similarly, Zhang et al. confined Sn_4_P_3_ particles within N‐doped carbon fibers (Sn_4_P_3_@CFs) via the electrospinning method.^[^ ^74^ ^]^ Benefiting from the structural merits, Sn_4_P_3_@CFs exhibited a high reversible capacity of 403 mAh g^−1^ after 200 cycles at 50 mA g^−1^, and a capacity of 160 mAh g^−1^ after 1000 cycles at 500 mA g^−1^.

In addition to Sn_4_P_3_, other tin phosphides like SnP,^[^ ^208^ ^]^ SnP_3_,^[^ ^209^ ^]^ and SnP_0.94_^[^ ^210^ ^]^ were also used as the anodes of PIBs. For example, Verma et al. synthesized SnP_3_/super P composite through high‐energy ball milling method,^[^ ^209^ ^]^ and it was confirmed that SnP_3_ wrapped by amorphous carbon relieved the volume expansion and enhanced the electrical conductivity.

#### 5.2.2 Fe‐Based Phosphides

Owing to its high theoretical capacity, low price, and non‐toxicity,^[^ ^211^ ^]^ FeP has been investigated as the anode for PIBs. Similar to Sn‐based phosphides, Fe‐based phosphides should be hybridized with carbonaceous materials for their utilizations. For instance, Li et al. prepared FeP/super P composite and employed it as the anode of PIBs.^[^ ^66^ ^]^ Carbon matrix improved the electrical conductivity, and inhibited the volume expansion of FeP; thus, a high capacity of 182 mAh g^−1^ after 50 cycles at 50 mA g^−1^ was achieved, accompanying with a capacity retention of 63%.

**Figure 9:**
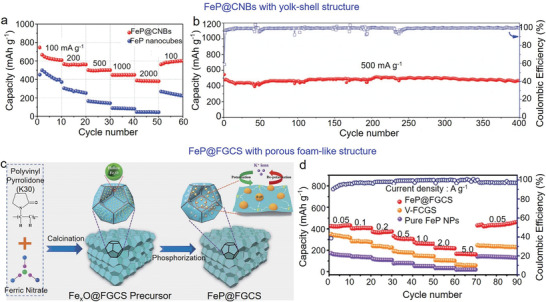
Fe‐based phosphides used in PIBs. a) Rate capability of FeP@CNBs and FeP nanocube under various current densities, and b) long‐term cyclic stability of FeP@CNBs at 500 mA g^−1^. Reproduced with permission.^[^ ^67^ ^]^ Copyright 2019, Wiley‐VCH. c) Schematic illustration of the fabrication of FeP@FGCS, and d) rate capability of PIBs when used FeP@FGCS as anode. Reproduced with permission.^[^ ^68^ ^]^ Copyright 2019, Royal Society of Chemistry.

Recently, new approaches for fabricating Fe‐based phosphides emerged. Particularly, Yang et al. synthesized yolk–shell FeP@carbon nanoboxes (FeP@CNBs) through the phosphorization of Fe_2_O_3_ nanoboxes.^[^ ^67^ ^]^ This structure restricted FeP nanoparticles within carbon shells, which improved the electronic conductivity, the generated voids and spaces between carbon shells and FeP nanoparticles alleviated volume expansion during cycles. As a result, a high initial capacity of 545 mAh g^−1^ was obtained, and the capacity maintained at 476 mAh g^−1^ after 400 cycles at 500 mA g^−1^ (**Figure** **9**a,b). Similarly, FeP nanoparticles were prepared to combine with graphene frameworks. For instance, Tan et al. used 3D foam‐like graphene as carbon scaffold (FGCS) to load FeP nanoparticles, generating a FeP@FGCS composite (Figure 9c).^[^ ^68^ ^]^ The FeP nanoparticles were tightly connected with FGCS through the strong P‐C bonds, improving the structural stability, whereas the special foam‐like structure increased the electron/ion transport. Consequently, the as‐prepared anodes exhibited good rate capability (164 mAh g^−1^ at 5.0 A g^−1^) and great long‐term cyclic stability (183 mAh g^−1^ after 1000 cycles at 3.0 A g^−1^) (Figure 9d).

#### 5.2.3 Co‐Based Phosphides

**Figure 10:**
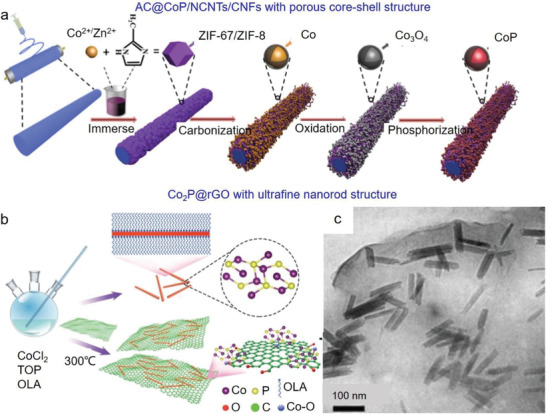
Co‐based phosphides used in PIBs. a) Schematic illustration of the synthesis of AC@CoP/NCNTs/CNFs composite. Reproduced with permission.^[^ ^213^ ^]^ Copyright 2019, Elsevier. b) Schematic illustration of the fabrication of ultrafine Co_2_P@rGO composite, and c) the typical morphology. Reproduced with permission.^[^ ^214^ ^]^ Copyright 2019, The Royal Society of Chemistry.

CoP with high theoretical capacity has attracted significant attention in PIBs, but they still experienced considerable volume expansion and low electronic conductivity. For instance, Liu et al. synthesized CoP@carbon spheres through the simple carbonization and phosphorization processes.^[^ ^212^ ^]^ This hollow structure enhanced the contact between active material and the electrolyte, and provided enough space for volume changes during cycles, whereas carbon layers on CoP nanoparticles increased the electronic conductivity and ensured the high structural integrity. To increase the capacity during long‐term cycles, Miao et al. designed a special and complex structure comprising amorphous carbon@CoP/N‐doped CNTs/CNFs (AC@CoP/NCNTs/CNFs) through a high‐temperature phosphorization process (**Figure** **10**a).^[^ ^213^ ^]^ As a result, an excellent rate performance (292 mAh g^−1^ at 3.2 A g^−1^) and ultra‐long cyclic stability (250 mAh g^−1^ after 1000 cycles at 0.8 A g^−1^) were achieved simultaneously.

Additionally, Co_2_P^[^ ^214^ ^]^ has been reported in PIBs. For example, Wang et al. prepared ultrafine Co_2_P nanorods on rGO (Figure 10b).^[^ ^214^ ^]^ The TEM image in Figure 10c confirmed the nanorod‐like morphology. Owing to the exposure of abundant active sites and enhanced electronic conductivity, the as‐prepared Co_2_P@rGO anode exhibited a high reversible capacity of 374 mAh g^−1^ at 20 mA g^−1^, and a high capacity retention of 64.7% after 200 cycles at 200 mA g^−1^.

#### 5.2.4 Mo‐Based Phosphides

**Figure 11:**
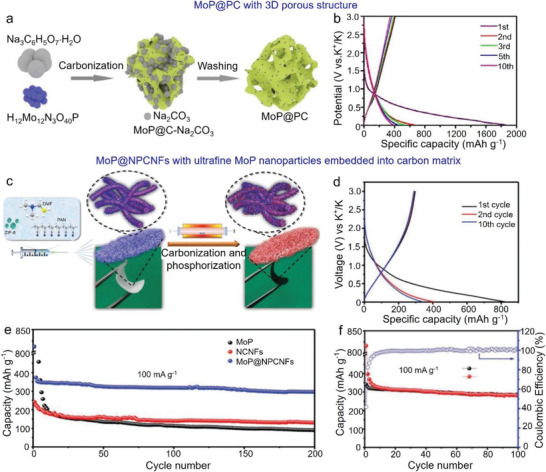
Mo‐based phosphides used in PIBs. a) Schematic illustration of the synthesis of MoP@PC, and b) galvanostatic charge/discharge profiles of MoP@PC at 100 mA g^−1^. Reproduced with permission.^[^ ^215^ ^]^ Copyright 2020, Elsevier. c) Schematic illustration of the synthesis of flexible and free‐standing MoP@NPCNFs membrane. d) Typical charge/discharge profiles and e,f) cyclic performance of MoP@NPCNFs. Reproduced with permission.^[^ ^69^ ^]^ Copyright 2020, Wiley‐VCH.

Molybdenum phosphides can also be used as the anodes of PIBs. Typically, Yan et al. synthesized MoP@PC composite by embedding molybdenum phosphide nano‐dots within petal‐like porous carbon (PC) (**Figure** **11**a).^[^ ^215^ ^]^ This structure provided numerous active sites and effectively alleviated the volume change during cycles; thus, a high reversible capacity of 330 mAh g^−1^ after 100 cycles (Figure 11b) was obtained.

Recently, Yi et al. prepared a flexible and free‐standing MoP@NPCNFs material using the electrospinning method, of which ultrafine MoP nanoparticles were uniformly planted within N/P co‐doped CNFs (NPCNFs) (Figure 11c,d).^[^ ^69^ ^]^ The ultrafine MoP nanoparticles supplied numerous active sites for K^+^ storage, N/P‐co‐doping characteristic improved the electrode wettability and ion diffusion, and 3D CNFs suppressed volume expansion and improved electronic conductivity. Consequently, MoP@NPCNFs exhibited a capacity of 280 mAh g^−1^ after 200 cycles at 100 mA g^−1^ and high CEs (Figure 11e,f). Similarly, Wang et al. prepared ultrasmall MoP nanoparticles within N/P‐co‐doped hollow CNTs.^[^ ^216^ ^]^ Electrochemical testing revealed a high specific discharge capacity of 255 mAh g^−1^ after 120 cycles at 1.0 A g^−1^.

#### 5.2.5 Other Phosphides

Besides the above‐discussed phosphides, other metal phosphides such as GeP,^[^ ^76^ ^]^ Se*_x_* P,^[^ ^217^, ^218^ ^]^ and Ni—Fe—P,^[^ ^219^ ^]^ have been explored as the anodes of PIBs. For instance, Zhang et al. prepared layered GeP_5_ as the anode material.^[^ ^76^ ^]^ It was revealed that potassium bis(fluorosulfonyl)imide salt helped to form a stable SEI layer, reducing the side reactions and further improving the cyclic performance, which can be attributed to the synergistic effect between K—P and K—Ge. Moreover, high electrochemical result was also achieved by using Se—P—carbon composite as the anode.^[^ ^218^ ^]^

In summary, phosphorus‐ and phosphide‐based materials have been widely investigated as the anodes of AIBs. According to the above discussions, pure phosphorus and phosphides are not suitable for serving as the anodes of AIBs, because of the structural collapse and low electronic conductivity during electrochemical cycles. Hybridizing phosphorus or phosphides with carbonaceous materials should be the promising solution. Of them, carbonaceous materials with 3D porous/hollow/yolk‐shell structures were verified to be the best candidates, owing to the diverse structural merits. 1) Carbonaceous materials acted as the highly conductive networks for promoting fast electron transfer, ensuring high reaction kinetics; 2) The sufficient spaces efficiently alleviated the volume expansion of phosphorus‐ and phosphide‐based materials during cycles; 3) The sufficient voids and channels shortened the diffusion length of alkali metal ions; and 4) The combination of phosphorus‐ and phosphide‐based materials with carbonaceous materials improved the entire structural integrity of electrode. The synergistic effects of these merits contributed to the high electrochemical performance of AIBs. Nevertheless, this strategy also encountered several shortcomings, including the low content of phosphorus‐ and phosphide‐based materials in electrodes, limited production scale, and complicated preparation processes, which needed to be addressed in the following research.

## 6 Phosphorus‐ and Phosphide‐Based Materials for Multivalent Metal‐Ion Batteries

In addition to single‐valent metal‐ion batteries (e.g., LIBs, SIBs, and PIBs), multivalent metal‐ion batteries such as magnesium‐ion batteries (MIBs) and calcium‐ion batteries (CIBs) using phosphorus‐ and phosphide‐based materials, were also emerged recently. However, the utilization of phosphorus‐ and phosphide‐based materials in multivalent metal‐ion batteries is in the initial stage. The relative works are introduced briefly in the following parts.

### 6.1 Phosphorus‐ and Phosphide‐Based Materials for MIBs

Recently, MIBs have attracted increasing attention, because of their diverse advantages including high theoretical capacity, low reduction potential, and low cost.^[^ ^220^ ^]^ MIBs have the following advantages: 1) MIBs usually have higher theoretical capacities than single‐valent metal ion batteries; 2) The ion diffusion is fast, because of the smaller radius of Mg^2+^ (0.072 nm); 3) MIBs have almost no dendrite growth behavior, and thus they are relatively safe and stable.^[^ ^221^ ^]^ The synergistic effects of these above merits demonstrated the promising applications of MIBs.

BP has recently been explored and adopted in MIBs, and the interlayer distance is large enough for the insertion of Mg^2+^.^[^ ^222^ ^]^ For instance, Jin et al. used density functional theory (DFT) to study the adsorption, diffusion and stability of Mg^2+^ on BP monolayer.^[^ ^223^ ^]^ The adsorption energy of Mg^2+^ on BP monolayer is −1.09 eV, and experimental results confirmed the high stability of Mg—P structure. Moreover, Banerjee et al. studied the effect of Mg—P bond on the electrochemical performance of MIBs.^[^ ^224^ ^]^ A synergistic effect between Mg—P bonds was found, thereby reducing the polarization of Mg^2+^. Through the first‐principle calculations, phosphorus was predicted to exhibit a low potential (0.15 V) and high specific capacity (1730 mAh g^−1^), when used as the anode of MIBs. This was also confirmed by the DFT calculations conducted by Sibari and co‐workers.^[^ ^225^ ^]^

### 6.2 Phosphorus‐ and Phosphide‐Based Materials for CIBs

Although calcium has many advantages, such as stable valence state, small ionic radius, and reserve abundant in the earth, CIBs still encountered various crucial issues, such as poor reversibility/recyclability, low working voltage, and serious polarization.^[^ ^226^, ^227^ ^]^ Recently, Muhammad et al. synthesized a 3D porous RP‐graphdiyne (3D‐PGDY) composite as the anode of CIBs.^[^ ^200^ ^]^ This unique structure provided abundant active sites and exhibited a low diffusion energy barrier, thereby a high Ca^2+^ storage capacity reaching 2129 mAh g^−1^ and a small volume change (2.32%) upon charging were achieved. This work paves the way to investigate CIBs using phosphorus‐based materials and promote the further practical utilization.

## 7 Conclusions and Perspectives

Phosphorus‐ and phosphide‐based materials with high theoretical capacity and long‐life cyclic stability have been considered as promising anodes of AIBs. However, phosphorus‐ and phosphide‐based materials in AIBs have low electrical conductivity, significant volume expansion, and slow reaction kinetics, which severely restricted their practical applications. To solve these issues, various promising solutions have been explored and adopted, and progress has been already achieved. This review summarizes the recent progress on AIBs using phosphorus‐ and phosphide‐based materials. Representative works have been listed and introduced, and the working principles of phosphorus‐ and phosphide‐based materials were discussed. However, some issues/challenges still existed, and more efforts are required in the following research:

1. The accurate working mechanisms of AIBs need to be investigated and verified. Although the working mechanisms of LIBs and SIBs have been clarified, those of other AIBs such as PIBs and multivalent metal‐ion batteries, are still lacking, owing to the different intrinsic physicochemical properties of alkali metal ions. Moreover, the investigations on multivalent metal‐ion batteries locate at the theoretical prediction stage, and experimental works are crucial to confirm the theoretical simulations. By valuing the advantages and disadvantages of these AIBs, SIBs would be the potential energy‐related devices for further applications, because of the high electrochemical performance, safe working conditions, reserve abundance, and evenly distribution of Na, and low costs.
2. The large‐scale production of phosphorus‐ and phosphide‐based materials with elaborately designed structures should be explored. To date, the emerged approaches for preparing phosphorus‐ and phosphide‐based materials included ball milling, hydrothermal, solvothermal, vaporization‐condensation, and others. However, these approaches have their own intrinsic limits, such as complex fabrication procedure, high costs, and low yield, restricting their further commercialization. Moreover, the desired micro‐and nanostructures with high content of phosphorus‐ and phosphide‐based materials (≥80 wt.%) are still lacking. The high content of phosphorus‐ and phosphide‐based materials in electrode would contribute to high capacity and energy density of AIBs. To resolve these pressing issues, the template method and spray‐drying method should be the promising solutions, and the combination of different approaches should be another effective strategy, which could avoid the drawbacks induced by one single approach.
3. Further improvement of electrochemical performance, especially the high rate capability, is still necessary for further applications. Currently, the electrochemical performance of AIBs still restricted by the low CE, slow diffusion kinetics, low electronic conductivity, and unstable structures. With the rapid increase of practical demands, significantly enhanced electrochemical results are still in urgent necessary. The promising solutions for addressing these problems include the hybridization with other active materials, surface/structure/morphology engineering, and heteroatom doping. For instance, besides carbonaceous materials, other active components, such as metals,^[^ ^228^ ^]^ and metal oxides,^[^ ^229^, ^230^, ^231^, ^232^ ^]^ have already been developed to hybridize with phosphorus, and achieving obviously enhanced electrochemical performance. Since rarely attention has been paid to these aspects, much effort is needed in the upcoming research.
4. Advanced techniques, such as in/ex situ, spectroscopical, and modern characterization tools, should be conducted to elucidate the actual electrochemical reactions of phosphorus‐ and phosphide‐based anodes in AIBs. Because the working principles of diverse AIBs are still missing, it is necessary to discover the electrochemical behavior of active components during charge/discharge cycles. For instance, it is difficult to figure out the accurate contribution percentages of different working mechanisms for diverse AIBs, since it provides new insights for designing and constructing promising electrodes. Moreover, by varying the working atmosphere of AIBs, the anode may experience distinctive working pathways and conversions. For instance, electrolyte systems may influence the volume expansion and generated intermediates of phosphorus‐ and phosphide‐based anodes during electrochemical reactions.^[^ ^233^, ^234^ ^]^ Notably, AIBs may not work when adopted phosphorus‐ and phosphide‐based materials in aqueous electrolytes, since these materials can react with water (causing fire and explosion), and electrocatalyze the water splitting under working voltage.
5. Theoretical simulations should be conducted to predict the electrochemical activity of phosphorus‐ and phosphide‐based anodes. To date, the extensively investigated phosphides include Fe‐, Co‐, Ni‐, Mo‐, and Sn‐based phosphides, while other phosphide types are rarely examined. Thus, it is necessary to adopt the theoretical studies like DFT and first‐principle calculations to explore the possibility of utilizing other phosphides in AIBs. Moreover, machine learning and artificial intelligence,^[^ ^235^ ^]^ as the new advanced study platforms, can boost the research and development of novel materials and battery systems, including electrode materials, separators, and electrolytes. The combination of these tools would undoubtedly accelerate the development of energy‐related devices. Thereafter, corresponding experiments should be conducted to confirm the predictions.
6. Considering the practical applications, the coupling of phosphorus‐ and phosphide‐based anodes with cathodes, electrolytes and separators should be well optimized. In addition to the anodic side, other components of AIBs affected the final electrochemical performance significantly. For instance, phosphate polyanion materials,^[^ ^236^, ^237^ ^]^ which usually served as the cathodes of AIBs because of the high working voltage window, determined the specific capacities and energy densities of full cells. Moreover, the current testing parameters and atmospheres are impractical, and they should be reset to cater the practical applications. New battery types, such as soft‐package, punch cells, and cable‐like cells, may need to be assembled and tested, which can be further utilized in special electronics. Meanwhile, to avoid the structural damage and separation from collectors of active species, it is necessary to intercalate them together using CNTs and/or CNFs. In addition, to satisfy the practical demands of high‐end electronics, phosphorus/carbon and Sn‐based phosphide/carbon composites with the merits of easy preparation and high electrochemical performance should be the potential candidates serving as anodes.

In summary, phosphorus‐ and phosphide‐based materials with various merits have been extensively used in AIBs. Although great progress has been already achieved in recent years, the development of PIBs and multivalent metal‐ion batteries are not mature, and they deserve more effort. Considering the rapid development and well understanding of their working mechanisms, the practical applications of high‐performance AIBs will be finally realized.

## Conflict of Interest

The authors declare no conflict of interest.
